# Association between sense of control and all-cause mortality: a prospective cohort study

**DOI:** 10.3934/publichealth.2025021

**Published:** 2025-03-20

**Authors:** Ying Li, Yilin Chen, Xiwen Ding, Yin Chen, Wei Jiang

**Affiliations:** 1 Department of Social Medicine, School of Public Health, Zhejiang University, Hangzhou, China; 2 School of medicine, Zhejiang University, Hangzhou, China; 3 College of Control Science and Engineering, Zhejiang University, Hangzhou, China

**Keywords:** sense of control, elderly people, mortality risk, prospective study, public health

## Abstract

The salutary effects of a sense of control on health are well acknowledged; however, rigorous studies evaluating its effect on mortality remain scarce. This study aimed to study the association between a sense of control and mortality and to identify the effect of changes in this sense of control on mortality risk. This prospective cohort study included 22,793 participants over age 50 and their spouses, drawn from a nationally representative U.S. sample from 2006 to 2018. Cox proportional hazard regression analyses estimated the association between sense of control and all-cause mortality. Kaplan-Meier survival curves were compared using the log-rank test, and changes in sense of control levels associated with mortality risk were evaluated using the Cox proportional hazards model. Over the 12-year follow-up period, 5027 deaths were recorded. An increased sense of control was significantly associated with decreased mortality risk, as revealed by stratified analysis according to sex and age. Hazard ratios (HRs) for the second, third, and fourth quartiles of sense of control levels were 0.91 (95% CI, 0.84–0.98), 0.83 (95% CI, 0.77–0.92), and 0.79 (95% CI, 0.72–0.87), respectively, relative to the first quartile. Compared to individuals with stable sense of control scores from baseline to study conclusion, the HR was 0.58 (95% CI, 0.48–0.70) for those with increased scores and 1.81 (95% CI, 1.53–2.13) for those with decreased scores. High levels of sense of control were significantly associated with reduced mortality risk. These findings underscore the importance of a sense of control as a focus for public health interventions.

## 1. Introduction

Amidst the global increase in the elderly population, there has been a rapid increase in the prevalence and mortality of age-related diseases [1]. Certain clinical ailments, a compromised physical well-being, and reduced life expectancy have been linked to the pathophysiology of mental disorders and have garnered increasing attention in recent years [2],[3]. Aging can result in adverse changes in physical and mental health [4],[5]. Older adults frequently grapple with comorbid mental disorders such as depression, dementia, and dependency, rendering their mental health challenges more complex than those faced by other age groups. Furthermore, mental disorders may be directly associated with increased mortality [6]–[8]. Over the past two decades, the prevalence of mental disorders has risen from 20% to approximately 33% among older adults in certain countries [9]. The escalating prevalence of mental disorders has placed greater demands on public health services, presenting substantial challenges in terms of treatment, interventions, and management.

While mental disorders can manifest at any stage from childhood to old age, certain periods in an individual's lifespan may entail a heightened risk of developing specific mental disorders [10]. Older adults often face increased challenges and adversities following retirement, including functional decline, shifts in social networks, bereavement due to the loss of spouses, and encounters with other stressors [11],[12]. The co-occurrence of mental disorders not only signifies more severe symptoms but also implies more formidable treatment complexities. Nevertheless, studies have indicated that many older adults strive to maintain their independence and well-being through readily accessible psychological resources [13]. Robust evidence supports the notion that positive attitudes and self-management can effectively reduce comorbidity in mental disorders among older adults.

The concept of “sense of control”, denoting an individual's belief in their capacity to influence or predict external events and guide the course of current or future occurrences, has gained increasing recognition as a crucial psychological resource throughout the human lifespan [14]. A strong sense of control emerges as a vital determinant of successful aging, particularly in older adults [15],[16]. Research has underscored the significant influence of a sense of control on enhancing an individual's psychological and social adaptation as well as their physical and mental well-being [17],[18]. Moreover, it may be a pivotal predictor of mortality [19],[20]. A study examined the health locus of control in relation to mortality using survival analysis in a prospective cohort study [21]. Subsequent investigations suggested that psychological and social interventions can effectively shape and enhance an individual's sense of control [22]. These findings highlight the potential of a sense of control as a significant avenue for improving health, thus sparking widespread interest among researchers.

While the benefits of a sense of control on individual physical and mental health have been well documented, there remains a paucity of studies exploring its association with severe clinical results. Some studies have endeavored to elucidate the link between a sense of control and mortality among older adults [23],[24]. Unfortunately, these findings have not been particularly encouraging because of limitations in study design, sampling methods, analytical approaches, and measurement instruments in prior research. The effects of implementing interventions aimed at enhancing a sense of control on health results and anticipated health benefits have not yet been comprehensively addressed. Consequently, our objective was to rigorously assess the connection between a sense of control and mortality while investigating the potential implications for public health through a robust prospective cohort study spanning a 12-year follow-up period. This endeavor aims to provide vital evidence for further research and development of intervention strategies.

## 2. Materials and methods

### 2.1. Study design and population

This study utilized data from the Health and Retirement Study (HRS), a nationally representative cohort survey initiated in 1992 that included the general population over the age of 50 in the United States, employing a multistage probability sampling design. The sample also included their spouses or partners regardless of age. The HRS was conducted biennially, amassing 14 waves of data up to 2018. Notably, a psychosocial and social health survey, an integral component of the HRS, was conducted in 2006. Psychosocial data were collected using self-administered questionnaires completed by 33,195 participants. The response rate, as evaluated among participants who had undergone a face-to-face core interview for the HRS in 2006, was approximately 90%. For the present study, individuals ineligible for self-administered questionnaires and those with incomplete data pertaining to the sense of control measurements were excluded. All participants provided written informed consent, and the study was approved by the institutional review boards of both the University of Michigan and Columbia University.

### 2.2. Measures

#### 2.2.1. Sense of control

Sense of control was assessed using two subscales consisting of 10 items [25],[26]. Personal mastery was gauged using the following questions: *I can do just about anything I really set my mind to. When I really want to do something, I usually find a way to succeed at it. Whether or not I am able to get what I want is in my own hands. What happens to me in the future mostly depends on me. I can do the things that I want to do*.

Additionally, five items were employed to measure perceived constraints: *I often feel helpless in dealing with the problems of life. Other people determine most of what I can and cannot do. What happens in my life is often beyond my control. I have little control over the things that happen to me. There is really no way I can solve the problems I have*.

Each item featured response options aligned with a 6-point Likert scale, ranging from “strongly agree” to “strongly disagree”. Notably, the perceived constraint items were reverse-coded. Subsequently, the total score and mean were computed across all 10 items to derive the individual sense of control scores. These scores were calculated for all seven waves, with higher scores indicating elevated levels of sense of control. The Cronbach's α coefficients were 0.86 for constraint and 0.89 for personal mastery, respectively.

#### 2.2.2. Covariates

All covariates available in the surveys were assessed during each wave, from 2006 to 2018. The traditional covariates included age in the survey year and sex (men and women). Additionally, the empirical covariates included race (white, black, or other), marital status (not married or married), educational level categorized by years (0–9, 10–12, over 13, or other), smoking status [whether the respondent reported smoking at all nowadays (yes or no)], alcohol use [whether the respondent reported having consumed an alcoholic drink in the past three months (yes or no)], and levels of physical activity (vigorous, moderate, or mild) [27]. Furthermore, candidate covariates were selected based on univariate analysis, including social security income (yes or no), number of resident children (0, 1, or ≥2), frequency of attending religious services (more than once a week, once a week, two or three times a month, one or more times a year, not at all, or other), self-rated health (excellent, very good, good, fair, poor, or other), and being bedridden (yes or no).

#### 2.2.3. All-cause mortality

All-cause mortality was defined by the International Classification of Diseases, 10th Revision, as the total number of deaths from any cause within a specific population over a given period.

#### 2.2.4. Outcomes

Eligible participants underwent continuous vital status surveillance to monitor the incidence of total mortality during each follow-up period. In addition, linkages to the National Death Index were established. In the event of a participant's death, surviving spouses, children, or other relevant individuals were interviewed to gather additional information. A sense of control assessment was also conducted during each follow-up period and at the end line.

### 2.3. Statistical analysis

We analyzed the self-assessed sense of control data obtained through leave-behind questionnaires from all participants during the follow-up period from 2006 to 2018. In total, 22,793 participants were included in the analysis. Sense of control scores were calculated if the participant responded to items of the sense of control measurement with no more than three missing values. Otherwise, the participants were excluded from the analysis. To provide an overview of participants' baseline characteristics, a descriptive analysis was conducted. Subgroups of characteristic variables were delineated by sex, and their representation was conveyed using frequency and percentage. The total scores of sense of control within the different age groups were computed at both the baseline and end line. SAS programming facilitated the visualization of score changes in the sense of control across age trajectories.

In the primary analysis, we employed Cox proportional hazard (CPH) models to estimate the association between sense of control and mortality incidence during the 12-year follow-up period. The proportional hazards assumption was assessed using graphical tests. Death status was identified and denoted as “1” in the CPH model as a terminal event. We calculated the person-years of follow-up for each participant, which included the time from the baseline survey to the point of death. Hazard ratios (HRs) and their corresponding 95% confidence intervals (CIs) were computed to assess mortality risk associated with varying levels of sense of control. Covariates, such as marital status, smoking status, alcohol use, physical activity, attendance of religious services, social security income, number of resident children, self-rated health, and bedridden status, were included in the CPH model. We also conducted a stratified analysis by sex and age to account for the effects of traditional confounding factors. A forest plot was used to present the HRs for the sense of control levels and covariates for mortality.

In an enhanced analysis, the sense of control scores were categorized into four quartiles based on univariate analysis. The first quartile (Q1) represents the 25th percentile (40 points), Q2 represents the median value (48 points), and Q3 represents the 75th percentile (55 points). Three dummy variables (DV) were created: DV1(Q1/Q2), DV2(Q1/Q3), and DV3(Q1/Q4), which were all referenced to the control group (Q1). HRs for mortality were calculated for these three dummy variables using the CPH regression analysis. We assessed the linear trend of sense of control levels in relation to mortality using a general linear model adjusted for race, marital status, education level, alcohol use, physical activity, attendance of religious services, social security income, number of resident children, self-rated health, and bedridden status. Survival curves for the four groups with different sense of control scores were plotted using the Kaplan-Meier method, and comparisons among all deaths across these groups were made using the log-rank test.

Furthermore, a sensitivity analysis was conducted to evaluate the causal association between changes in the sense of control levels and mortality during the follow-up period using CPH regression analysis. Changes in the sense of control scores were calculated based on the differences between the baseline and end-line survey responses. These changes were categorized into three groups: no change, higher than baseline score, and lower than baseline score. Two dummy variables were created relative to the no-change group, and comparisons among the three groups were visually presented. Statistical significance was indicated by two-tailed p-values lower than 0.05. All statistical analyses were performed using the SAS software (version 9.4, SAS).

### 2.4. Availability of data and materials

Data described in this paper are stored in the University of Michigan Open Research Data Platform, a public data repository https://hrs.isr.umich.edu/.

### 2.5. Ethics approval and consent to participate

All participants provided informed consent before participation. The study was approved by the institutional review boards of both the University of Michigan and Columbia University.

## 3. Results

**Figure 1.**
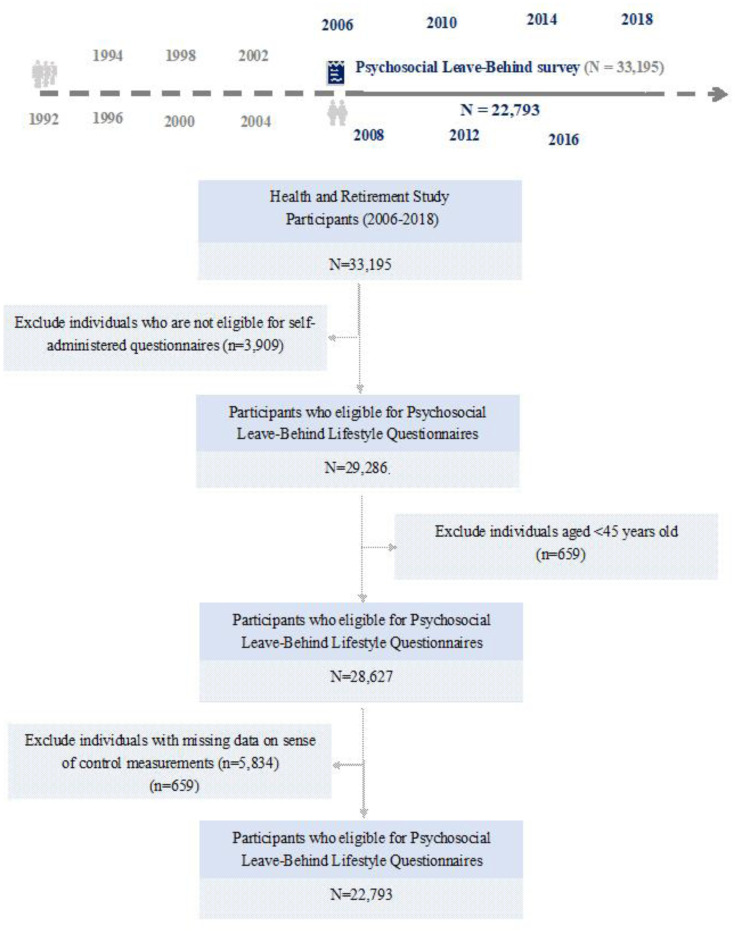
Flowchart of the participants and follow-up timeline.

The study settings are illustrated in Figure 1. During the 12-year follow-up period, which included 22,793 participants, 5027 deaths from all causes were identified. Among these, 9581 were women and 13,212 were men, with an average age at the time of death of 73.7 years.

Table 1 provides a summary of participants' baseline characteristics. Notably, 30.6% of participants were aged 45–55 years, whereas only 3.1% were aged 85 years and above. Approximately 74.6% of participants were identified as non-Hispanic whites and reported a relatively high level of education. Most participants did not reside with their children, and there was a higher proportion of unmarried individuals among women than among men.

**Table 1.** Baseline characteristics of participants in the study (2006–2018).

Variable	Men (N = 9581)		Women (N = 13,212)	
	n	%	n	%
Characteristic variables				
Age groups (years)				
45−54	2209	(23.1)	3571	(26.6)
55−64	3055	(31.9)	4125	(31.2)
65−74	2685	(28.0)	3262	(24.7)
75−84	1336	(13.9)	1708	(12.9)
85+	296	(3.1)	600	(4.6)
Race/ethnicity				
White	7294	(76.8)	9715	(74.0)
Black	1505	(15.8)	2514	(19.2)
Other	703	(7.4)	897	(6.8)
Marital status				
Non-married	2527	(26.4)	6020	(45.6)
Married	7054	(73.6)	7192	(54.4)
Education (years)				
0−9	1217	(12.8)	1465	(11.1)
10−12	3508	(36.6)	5596	(42.4)
13+	4667	(48.7)	5976	(45.2)
Other	189	(1.9)	175	(1.3)
Social security income				
Yes	4901	(55.9)	6584	(54.6)
No	3862	(44.1)	5476	(45.4)
Number of resident children				
0	6886	(71.9)	9100	(68.9)
1	1658	(17.3)	2812	(21.3)
≥2	1037	(10.8)	1300	(9.8)
Attends religious services				
More than once a week	1159	(12.1)	2289	(17.3)
Once a week	2128	(22.2)	3566	(27.0)
Two or three times a month	1258	(13.1)	1837	(13.9)
One or more times a year	2209	(23.1)	2634	(19.9)
Not at all	2816	(29.4)	2863	(21.7)
Other	11	(0.1)	23	(0.2)
Health rating				
Excellent	1175	(12.2)	1498	(11.3)
Very good	2873	(30.0)	3827	(29.0)
Good	2950	(30.8)	4106	(31.1)
Fair	1914	(20.0)	2773	(21.0)
Poor	662	(6.9)	998	(7.5)
Other	7	(0.1)	10	(0.1)

**Figure 2.**
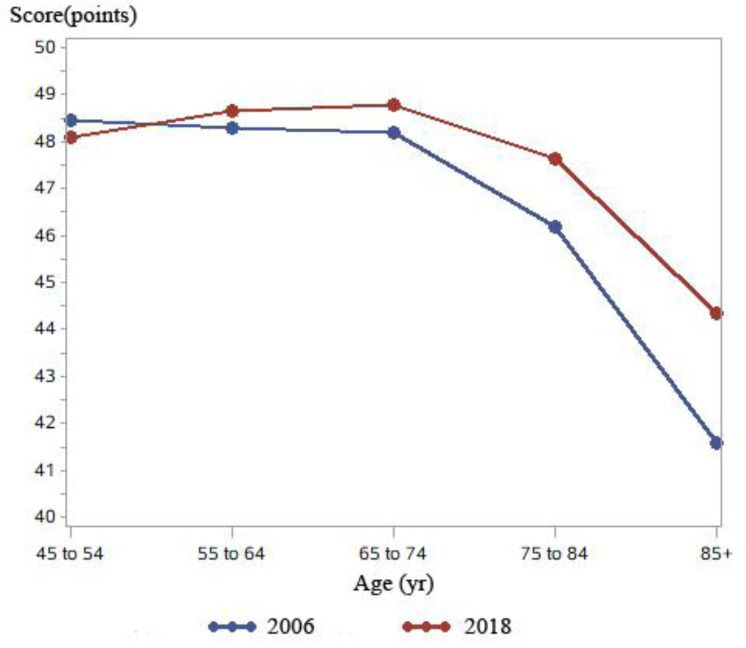
Sense of control scores by age group (2006–2018).

Figure 2 shows the sense of control scores categorized by age group. These scores exhibited an upward trend for each age group in 2018 compared with the sense of control scores in 2006. However, it is noteworthy that the 45–54 age group was an exception, with slightly lower scores in 2018. In 2006, there was a declining trend in the sense of control scores with increasing age. In 2018, the sense of control scores showed an increasing trend for the age group of 45–54 years and a decreasing trend for the age group of 65–74 years. In the 75–84 years age group, the sense of control scores sharply declined with increasing age, reaching their lowest points in the group aged 85 years and above.

**Figure 3.**
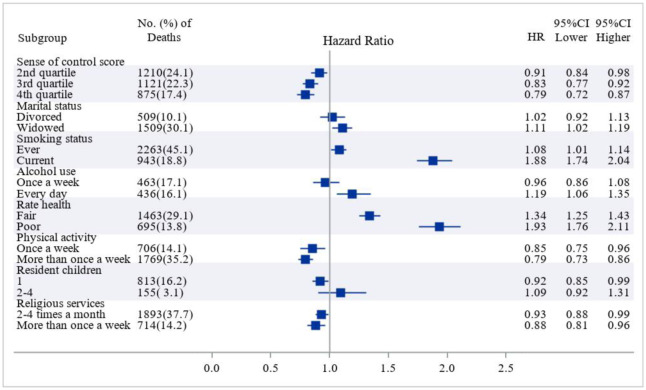
Hazard ratios of sense of control and related factors for all-cause mortality (2006–2018).

Table 2 presents the results of time-to-event analysis using the CPH model. During the follow-up period from 2006 to 2018, 189,213.2 person-years were observed for all participants, with 33,086.2 person-years observed for participants who had deceased. In the fully adjusted model, higher levels of sense of control were associated with a reduced risk of all-cause mortality, with an HR of 0.98 (95% CI, 0.97–0.99, p < 0.001) for each one-point increase in the score. Among those who were married, engaged in alcohol use, participated in physical activity, attended religious services, and had resident children, there was a reduced risk of mortality [HR, 0.92 (95% CI, 0.86–0.98), 0.85 (95% CI, 0.80–0.90), 0.72 (95% CI, 0.67–0.77), 0.85 (95% CI, 0.79–0.90), and 0.89 (95% CI, 0.83–0.96), respectively]. Conversely, among those who smoked, reported poor health, were bedridden, and had social security income, there was an increased risk of mortality, with HRs of 1.52 (95% CI, 1.43–1.63), 1.76 (95% CI, 1.65–1.87), 1.02 (95% CI, 1.01–1.03), and 1.41 (95% CI, 1.25–1.59), respectively. The detailed subgroup analysis results are shown in Figure 3.

**Figure 4.**
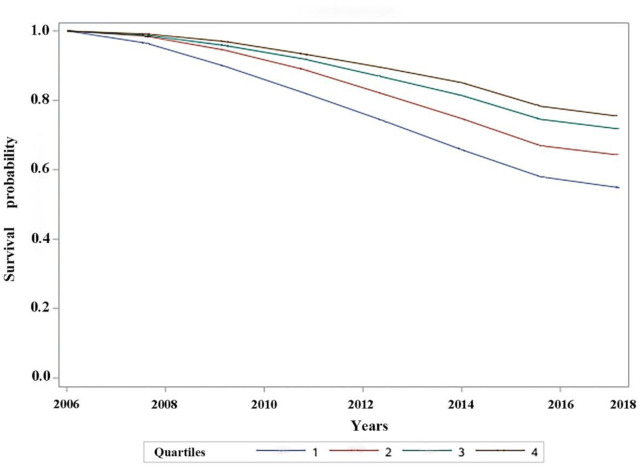
Probability of survival in the group with different levels of sense of control (2006–2018).

Table 3 presents the findings of the enhanced analysis, highlighting the association between the sense of control and all-cause mortality. Higher levels of sense of control were significantly associated with a reduced risk of mortality, displaying a strong dose-response relationship across different groups. Compared with individuals with low levels of sense of control (≤40 points), those with sense of control scores of 41–48 points, 49–55 points, and ≥56 points had HRs of 0.91 (95% CI, 0.84–0.98), 0.83 (95% CI, 0.77–0.90), and 0.79 (95% CI, 0.72–0.87), respectively. A significant linear trend was observed (p < 0.001). Figure 4 illustrates the survival function, demonstrating a lower cumulative incidence of mortality in the group with higher levels of sense of control than in the group with lower levels.

**Table 2.** Hazard ratios of sense of control and related factors for all-cause mortality (2006–2018).

Characteristics	N	Events death, n	Person-years	HR (95% CI)	p-value
Sense of control scores (points)					
7–60	22,793	5027	189,213.2	0.98 (0.97−0.99)	<0.001
Marital status					
Non-married	8547	2281	66,802.4	1.00 (Reference)	
Married	14,246	2746	122,410.8	0.92 (0.86−0.98)	0.006
Smoking status					
No	8142	1808	83,844.1	1.00 (Reference)	
Yes	9516	3206	105,350.6	1.52 (1.43−1.63)	<0.001
Alcohol use					
No	9670	2740	82,096.8	1.00 (Reference)	
Yes	13,118	2286	107,074.1	0.85 (0.80−0.90)	<0.001
Physical activity					
No	4272	1639	32,546.8	1.00 (Reference)	
Yes	18,508	3382	156,592.1	0.72 (0.67−0.77)	<0.001
Attended religious services					
No	10,522	2415	84,049.2	1.00 (Reference)	
Yes	12,237	2607	104,970.8	0.85 (0.79−0.90)	<0.001
Social security income					
No	9338	658	83,946.1	1.00 (Reference)	
Yes	11,485	4351	102,159.3	1.41 (1.25−1.59)	<0.001
Number of resident children					
0	15,986	4058	136,418.6	1.00 (Reference)	
≥1	6807	969	52,794.6	0.89 (0.83−0.96)	0.002
Rate health					
Good	16,429	2862	141,861	1.00 (Reference)	
Bad	6347	2158	48,214.7	1.76 (1.65−1.87)	<0.001
Bedridden					
No	19,829	4358	166,335.6	1.00 (Reference)	
Yes	2899	628	22,515.4	1.02 (1.01−1.03)	<0.001
Other					

**Table 3.** Hazard ratios of sense of control for all-cause mortality by quartile and linear trend test (2006–2018).

Sense of control scores by quartile (points)	N	Events death, n	Person-years	HR (95% CI)	p-value
Quartile (Q1–Q4)					
≤40	5702	1821	44,755.9	Reference	
41–48	5093	1210	42,010.5	0.91 (0.84−0.98)	0.009
49–55	6173	1121	52,605.1	0.83 (0.77−0.90)	<0.001
≥56	5825	875	49,841.7	0.79 (0.72−0.87)	<0.001
				*P* for trend	<0.001

Note: Adjusted for race/ethnicity, marital status, education levels, physical activity, smoking status, alcohol use, attendance of religious services, social security income, number of resident children, health rating, and bedridden condition.

**Figure 5.**
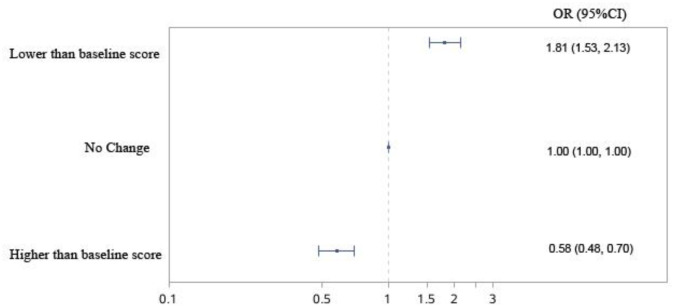
Altered levels of sense of control for mortality risk during the follow-up period (2006–2018).

In Table 4, sensitivity analysis confirms the causal association between changes in the sense of control levels and all-cause mortality during the follow-up period from 2006 to 2018. Among the participants, 15.5% showed no change in sense of control scores, 27.2% demonstrated an increase, and 57.3% displayed a decrease in sense of control scores between baseline and end-line surveys. After adjusting for all covariates, the group with an increase in sense of control scores had a significantly lower mortality risk, with an HR of 0.58 (95% CI, 0.48–0.70). Conversely, the group with a decrease in sense of control scores had a significantly higher mortality risk, with an HR of 1.81 (95% CI, 1.53–2.13). These changes in mortality risk are shown in Figure 5.

**Table 4.** Hazard ratios of changed senses of control levels for all-cause mortality during the follow-up period (2006–2018).

Change in sense of control scores	N	Events death, n	Person-years	HR (95% CI)	p-value
Baseline to end line (points)					
No change (0)	3529	261	22,463.2	Reference	
Higher than baseline score (1–46)	6194	555	59,526.0	0.58 (0.48−0.70)	<0.001
Lower than baseline score (1–50)	13,070	4211	107,224.0	1.81 (1.53−2.13)	<0.001

Note: Adjusted for race/ethnicity, marital status, education levels, physical activity, smoking status, alcohol use, attendance of religious services, social security income, number of resident children, health rating, and bedridden condition.

## 4. Discussion

In this national prospective cohort study, we found a significant association between the sense of control and the risk of all-cause mortality over a 12-year follow-up period. Even after adjusting for age and other covariates, it became evident that higher levels of sense of control were strongly associated with a reduced risk of mortality. This association follows a robust dose-response pattern. Furthermore, we bolstered our findings by identifying a causal association between changes in sense of control levels and corresponding changes in mortality risk over time.

The idea of a sense of control has garnered substantial attention from researchers across a multitude of fields [28],[29], including psychiatry, psychology, sociology, social psychology, behavioral science, gerontology, and management [30],[31]. Three decades ago, scholars began to contemplate the potential therapeutic value of restoring a patient's sense of control, particularly in the context of treating serious illnesses [32]. Within the domain of public health, which traditionally focuses on mitigating disease risk factors and mortality, it is important to identify the factors that can promote health. Of particular interest are potentially modifiable factors that have the capacity to not only curtail the growing prevalence of chronic conditions but also reduce healthcare costs.

Over recent decades, researchers have dedicated considerable efforts to exploring the connection between the sense of control and mortality [33]. However, evidence remains limited, and only a few studies have explored this relationship. In the nascent stages, the effect of a sense of control on health was just beginning to be acknowledged and studied, and a clear definition was lacking. For instance, one study assessed the association between a sense of control and mortality using a powerlessness instrument with four items [34]. Unfortunately, the reliability of this measure of sense of control was relatively low (alpha = 0.60). Moreover, the study primarily involved relatively young participants, with age ranges of 45–59 and 30–44 years for men and women, respectively. Consequently, an association between the sense of control and mortality was observed in men but not in women. More recently, a comprehensive study explored the effects of a sense of control on physical, psychological, and behavioral health and mortality among older adults [35]. Although this study included a sample of approximately 13,000 individuals, it had a relatively short observation period, comprising only a 4-year follow-up with measurements taken at pre-baseline and baseline, omitting a sense of control measurement at the end line. The assessment of mortality risk was based on a logistic regression model, neglecting time in the analysis. To date, these research limitations have not been addressed adequately.

In the present study, we addressed these shortcomings by including 22,793 participants and conducting a comprehensive 12-year follow-up study. We calculated the number of follow-up person-years for all participants, including those who died. Our evaluation of the association between sense of control and mortality risk not only employed the CPH model but also assessed the relationship between sense of control and survival time. Furthermore, we categorized the sense of control level into quartiles, confirming a dose-response relationship between the sense of control level and mortality. We also considered whether the intensity of the mortality changes was associated with a sense of control. To this end, we divided the change in the sense of control level into three groups based on baseline and end-line scores. Our findings revealed a reduced mortality risk in the group with an increased sense of control level from baseline to end line and, conversely, an increased mortality risk in the group with a reduced sense of control level. This robust evidence substantiates the causal link between a sense of control and mortality and offers valuable insights for the design and implementation of future interventions.

Studies have consistently suggested that high levels of sense of control are associated with positive physical and mental health among older adults [36]. Consequently, numerous researchers have endeavored to uncover the complex relationship between a sense of control and aging. Previous studies have often indicated that a sense of control exhibits a declining trend with advancing age [37]. Some smaller-scale studies have shown that the mean level of sense of control is relatively high in individuals aged 18–50 years and subsequently diminishes with age [38]. However, there are variations in these findings, with some studies suggesting that the sense of control in different domains may either increase or decrease with age [39]. For instance, the sense of control related to parental roles tends to decline with age, while it appears to be stronger in the financial domain for adults or older adults than for younger individuals [40].

In this study, we conducted an analysis to investigate the effect of age, period, and cohort on the sense of control. Our findings indicate a gradual decline in the sense of control with age among baseline participants aged 55–64 years in 2006. This result aligns with previous research, highlighting that changes in physical function, psychological well-being, and social status are significant factors that influence one's sense of control [41]. The period effect, which represents changes related to specific time periods, is noteworthy [42]. Interestingly, we observed that although the sense of control typically decreased with age, the sense of control among baseline participants in 2006 was significantly lower than that among end-line participants in 2018. This disparity may be attributed to socioeconomic development, which is known to positively influence an individual's sense of control. Compared with the sense of control level in 2006, we speculate that socioeconomic advancements contributed to an increased sense of control in 2018. The cohort effect, which reflects the interplay between external factors and individuals' internal development, has garnered attention. It is widely believed that the sense of control declines with age, primarily due to the lack of evidence from large-scale prospective cohort studies. However, our study revealed that while participants aged 65–74 years in 2006 mostly fell into the age group of 75–84 in the 2018 end-line survey, their sense of control scores did not significantly decline, except for those aged 85 years and above. Additionally, in 2006, participants in the age group of 65–74 years exhibited a slight decline in sense of control scores, but in 2018, participants in the same age group showed an increase in sense of control scores. This suggests that under the influence of individual and environmental factors, individuals experience an increasing trend in sense of control levels, with a gradual decline occurring at the age of 75 and above. Studies have proposed that economic independence, improved health insurance, social support and engagement, and physical well-being can effectively enhance an individual's sense of control [43],[44]. Our study confirmed the findings of previous qualitative studies.

The primary strength of our study lies in its large-scale national prospective cohort design, which enhances our understanding of the causal link between the sense of control and mortality. The HRS collected a wealth of data, including physiological, psychological, social, and biomarker variables, enabling us to comprehensively adjust for confounding factors when evaluating the association between sense of control and mortality. A notable advantage of our study is the ability to conduct stratified analyses by age and sex due to the ample sample size, allowing us to account for the potential effects of age and sex and accurately estimate the effect of sense of control on mortality. However, it is important to acknowledge the major limitation of this study. Not all participants were asked to complete a leave-behind questionnaire survey, which was administered selectively to some participants who had completed the core interview. In 2006, the response rate for leave-behind questionnaires was approximately 90%, while the overall response rate was approximately 74%. We intended to utilize sample weights for data analysis, but the 2006 sample weight was still in the developmental stage and unavailable for use. However, younger individuals capable of independently completing the questionnaire were selected to participate in the leave-behind survey. We minimized the selection bias associated with the left-behind survey as much as possible, through an age stratification analysis. Additionally, because this was a large-scale cohort study, multistage area probability and oversampling of racial minorities were incorporated into the initial research design to enhance representativeness, thereby significantly reducing sampling bias. Furthermore, we compared the general characteristics of the participants with those of individuals who did not participate in the study, and no significant differences were found. Therefore, the sample in this study can reasonably be considered representative of the general population of the United States. In addition, although the HRS contains a vast and rich dataset, it is not specifically designed to study the sense of control. Over time, to accommodate new content without overburdening respondents and maintain consistency across each wave of questionnaires, there may have been an insufficient exploration of content validity and the factors that influence the sense of control.

## 5. Conclusions

In conclusion, mortality serves as a traditional yardstick for gauging public health status, and the assessment of mortality risk is a fundamental component in shaping public health policies. In our study, we provided robust evidence supporting the causal relationship between the sense of control and mortality. With the rapid aging of the global population, there has been a surge in the prevalence of diseases and mortality, presenting substantial challenges to global public health infrastructure. Identifying health risk factors and estimating the relationship between exposure to these factors and health outcomes remains a top priority in the development of effective public health strategies. Our study underscores the significant role of a sense of control in reducing mortality risk and its pivotal implications for healthy aging within the domain of public health. Future research endeavors should focus on exploring modifiable factors that influence the sense of control, elucidating the pathways and mechanisms through which the sense of control affects social, physiological, and mental health.

## Use of AI tools declaration

The authors declare they have not used Artificial Intelligence (AI) tools in the creation of this article.
